# *Arthrospira platensis* Preserves Uterine Function by Modulating Electromechanical Coupling and Redox Pathways During Resistance Training in Female Rats

**DOI:** 10.3390/ijms262311440

**Published:** 2025-11-26

**Authors:** Bárbara Cavalcanti Barros, Anderson Fellyp Avelino Diniz, Francisco Fernandes Lacerda-Júnior, Petruska Pessoa da Silva Souza, Adriano Francisco Alves, Paula Benvindo Ferreira, Fabiana de Andrade Cavalcante, Bagnólia Araújo da Silva

**Affiliations:** 1 Postgraduate Program in Natural and Synthetic Products Bioactive/Health Sciences Center, Federal University of Paraiba, João Pessoa 58051-900, Brazil; barbaracavalcanti@ltf.ufpb.br (B.C.B.); lacerdafar17@gmail.com (F.F.L.-J.); adrianofalves@gmail.com (A.F.A.); paulabenvindo91@gmail.com (P.B.F.); fabianacavalcante@ltf.ufpb.br (F.d.A.C.); bagnolia@ltf.ufpb.br (B.A.d.S.); 2 Postdoctoral National Council for Scientific and Technological Development, Functional Pharmacology Laboratory, Federal University of Paraíba, João Pessoa 58051-900, Brazil; 3 Health Sciences Center, Federal University of Paraiba, João Pessoa 58051-900, Brazil; petruskapessoa@gmail.com; 4 Department of Biomedical Sciences, Health Sciences Center, Federal University of Paraíba, João Pessoa 58051-900, Brazil; 5 Pharmaceutical Sciences Department, Health Sciences Center, Federal University of Paraíba, João Pessoa 58051-900, Brazil

**Keywords:** progressive strength training, uterus, oxidative stress, *algae*, smooth muscle contractility, Spirulina

## Abstract

Algae-derived bioactives have emerged as promising nutraceuticals due to their ability to modulate key molecular pathways under physiological stress. *Arthrospira platensis* (Spirulina), a cyanobacterium widely recognized for its antioxidant and anti-inflammatory properties, is proposed as a functional supplement to preserve smooth muscle physiology. Progressive strength training (PST) can induce oxidative stress and disrupt electromechanical coupling in the uterus, potentially impairing female reproductive function. This study investigated whether supplementation with *A. platensis* prevents PST-induced uterine dysfunction and elucidated the molecular mechanisms involved. Virgin Wistar rats were divided into five groups: sedentary with saline (GS), sedentary with *A. platensis* (GAP100), adapted control (GC), PST-trained (GT), and PST-trained with *A. platensis* (GTAP100). An eight-week water-jump PST protocol was applied. Uterine contractile responses were recorded in isolated organ baths after cumulative KCl stimulation, in the absence or presence of pathway-specific inhibitors targeting nitric oxide synthase, cyclooxygenase, NADPH oxidase, or superoxide dismutase. Histological evaluations of uterine and ovarian tissues were also performed. PST increased contractile efficacy and myometrial thickness, associated with oxidative stress and activation of NO, COX, and NADPH oxidase pathways. Supplementation with *A. platensis* attenuated these alterations by enhancing NO signaling, stimulating relaxant prostanoids, and reducing superoxide production. These protective effects were abolished by inhibitors, confirming mechanistic involvement. Overall, our findings provide molecular evidence that *A. platensis* supplementation preserves uterine smooth muscle physiology under high-intensity resistance training, supporting its potential as a nutraceutical strategy for female reproductive health.

## 1. Introduction

Progressive strength training (PST) is a widely used exercise modality known to induce muscle hypertrophy, neuromuscular adaptations, and improvements in maximal strength [1,2,3]. Beyond these well-established benefits, PST also promotes metabolic adaptations, including increased glycolytic enzyme activity, elevated intracellular ATP and phosphocreatine levels, and reductions in mitochondrial content and capillarization. These effects vary according to training intensity, frequency, and individual factors such as age, sex, genetic background, and training history [4,5,6,7].

The female reproductive system is particularly sensitive to physiological stress. Intense PST may lead to dysfunctions such as delayed menarche, amenorrhea, and oligomenorrhea, reported in up to 79% of athletes [8,9,10,11]. This indicates that the uterine tissue may respond to metabolic and oxidative stimuli even without direct exposure to exercise. Moreover, PST increases the production of reactive oxygen species (ROS) and reactive nitrogen species (RNS), both of which play dual roles: modulating reproductive processes such as oocyte quality and implantation, but causing damage when produced in excess—depending on exercise intensity, conditioning, and nutritional status [6,7,8,9].

These reactive species also profoundly impact calcium-mediated uterine contraction mechanisms. Ca^2+^ release through channels such as the inositol trisphosphate receptor (IP_3_R) and the sensitivity of uterine smooth muscle to calcium are strongly influenced by redox states; under hypoxic conditions, ROS production increases via mitochondrial complexes I and III, compromising myometrial contractility [3,10,11,12,13,14,15,16,17]. Although progressive strength training (PST) does not directly stimulate uterine smooth muscle, systemic responses to intense and repetitive exercise—such as hormonal fluctuations, oxidative stress, and ischemia/reperfusion phenomena—can indirectly modulate uterine contractility. Repeated intense uterine contractions have been shown to cause transient ischemia and hypoxia, triggering adaptive or pathological responses [18]. In addition, oxidative stress is strongly implicated in female reproductive dysfunction [19,20] and intense exercise is associated with altered gonadotropin release and menstrual irregularities [10]. Resistance exercise has also been shown to modify ovarian physiology [21,22], and activation of MAPK/ERK signaling has been linked to increased uterine contractility [23,24]. These mechanisms support the rationale that PST may indirectly affect uterine physiology.

In the uterus, repeated or intense contractions can transiently reduce uteroplacental blood flow, leading to local hypoxia and eliciting adaptive or pathological responses depending on the context. Experimental and clinical studies have shown that excessive uterine activity during labor is associated with transient ischemia of the myometrium and can modulate cellular signaling, cytokine expression, and contractile protein activity, which may be protective or, in some cases, deleterious to both maternal and fetal tissues [25,26,27,28]. These studies provide clear evidence that the intensity and frequency of uterine contractions are key determinants of tissue oxygenation and the subsequent cellular response.

This redox modulation of calcium-signaling pathways becomes particularly relevant in different physiological and pathological contexts within the uterus. For instance, during the menstrual phase, small myometrial contractions, associated with vasoconstriction of the endometrial spiral arteries, induce transient endometrial hypoxia—a physiological event that signals the end of menstruation [29]. Moreover, in pregnant animal models, moderate-to-intense exercise results in reduced uterine blood flow—estimated at 15–25% during moderate exercise and approximately 30% during prolonged efforts or late gestation—accentuating conditions of critical oxygenation [30]. Although this decrease in perfusion does not constitute complete ischemia, it can significantly alter the oxygen microenvironment, contributing to either adaptive or pathological responses depending on the duration and intensity of the redox/kinetic stimulus.

Furthermore, no previous studies have directly evaluated the impact of PST on uterine contractility. However, Ferreira et al. investigated this connection in an animal model (rats), assessing uterine reactivity after strength training with or without dietary supplementation. Their results demonstrated that PST increased contractile efficacy, both in pharmacomechanical coupling (using oxytocin) and electromechanical coupling (using KCl), while also elevating oxidative stress in the uterine tissue. These findings indicate that the uterus is a functional target of exercise and responds to the physiological stress of PST. Notably, when combined with *Arthrospira* (*Spirulina*) *platensis* supplementation, this effect was prevented, while the tissue’s antioxidant capacity was further enhanced [31].

**Figure 1:**
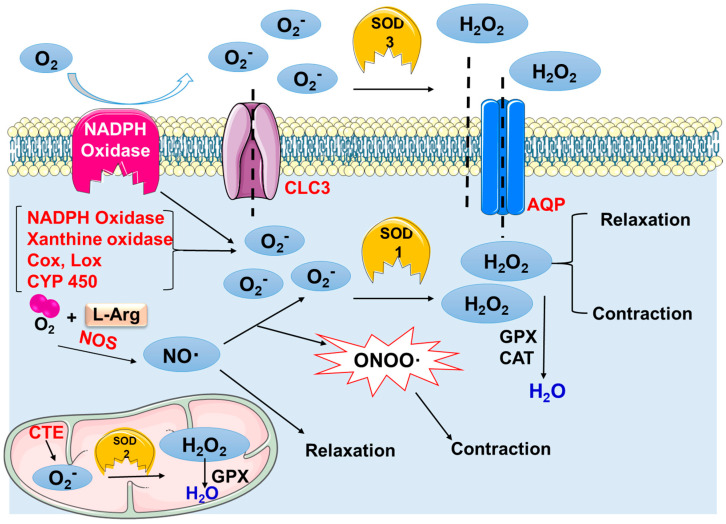
Schematic representation of redox signaling in mamma-lian cells. In aerobic organisms, diverse sources of superoxide (O_2_•^−^) production include the mitochondrial electron transport chain (ETC), NADPH oxidase, xanthine oxidase (XO), cytochrome P450 monooxygenases (CYP450), cyclooxygenase (COX), and lipoxygenase (LOX); in addition, nitric oxide synthase (NOS—e.g., NOS2) also contributes to ROS production and subsequent signaling. SOD catalyzes the dismutation of O_2_•^−^ to hydrogen peroxide (H_2_O_2_), which in turn can cross membranes via aquaporins (AQPs) or react internally, being neutralized by catalase (CAT) or glutathione peroxidase (GPx). Other components illustrated include chloride channels (CLC3), which allow superoxide anion entry, and cellular ROS-scavenging systems. This figure is for illustrative purposes only and does not reflect the original data from this study.

Additionally, it is well established that intense physical training, particularly when combined with inadequate recovery, can disrupt systemic redox balance and cause hormonal dysregulation. Such alterations compromise the homeostasis of the reproductive system, amplifying adverse effects through oxidative stress [1,3,4]. Thus, even though exercise does not act directly on the uterus as a local mechanical stimulus, several systemic mechanisms justify the relevance of the uterus in this experimental context (Figure 1). 

Our group has previously demonstrated the antioxidant and pharmacological potential of *Arthrospira platensis* in smooth muscle tissues, including the uterus and ileum [31,32]. This cyanobacterium protects against excessive contractile activity and also acts in other tissues such as the aorta, trachea, and corpus cavernosum, reinforcing its relevance in the modulation of smooth muscle tone [33,34,35,36,37].

Given the uterus’s unique contractile profile and its high sensitivity to oxidative stress, it is essential to investigate how PST affects electromechanical coupling mechanisms in this tissue. Although previous studies are limited, Ferreira et al. provided valuable evidence in a Wistar rat model subjected to PST with or without dietary supplementation, demonstrating that PST increased contractile efficacy (Emax) and reduced potency (pCE_50_) in response to KCl-induced stimulation. Notably, supplementation with *A. platensis* (50 and 100 mg/kg) prevented these alterations, with the 100 mg/kg dose yielding the most pronounced effects. These findings highlight the protective action of *A. platensis* and underscore the need to elucidate the molecular mechanisms involved in this response.

Most of the available evidence on exercise and female reproductive function derives from studies on high-intensity interval training (HIIT) or sustained excessive exercise. However, to our knowledge, there is no data specifying how progressive resistance training influences ovarian or uterine physiology. This lack of direct evidence highlights the novelty of our study and underscores the need to investigate whether the uterus is a functional target of resistance exercise.

This study should be viewed as exploratory and hypothesis-generating, providing an initial step toward understanding how resistance training affects uterine physiology and how *Arthrospira platensis* supplementation may mitigate these effects.

## 2. Results

### 2.1. Histomorphometric Evaluation of Uterine Tissue

**Figure 2:**
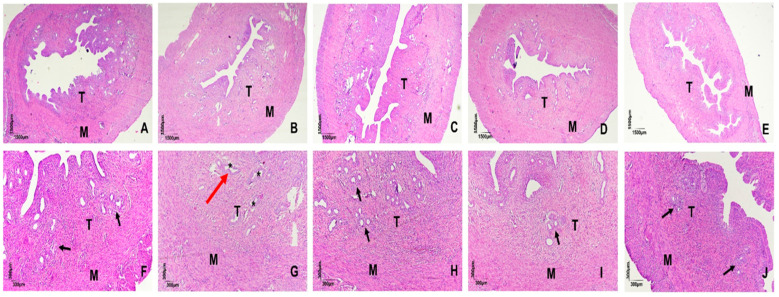
Photomicrograph of rat uterus. Effect of progressive strength training and supplementation with *Arthrospira platensis* on the morphology of the uterus of rats, in the groups GC (**A**,**F**), GT (**B**,**G**), GTAP100 (**C**,**H**), GS (**D**,**I**) and GAP100 (**E**,**J**). Endometrium (T) and myometrium (M); Circular and multiple glands (black arrows). Individualized endometrial glands with single and diffuse morphological distribution (* and red arrow).

Histological examination of uterine sections stained with hematoxylin and eosin revealed preserved endometrial morphology and circular glandular formations in the GC (control), GTAP100 (trained + AP), GS (saline), and GAP100 (saline + AP) groups. In contrast, GT (trained) animals exhibited disrupted endometrial architecture with individualized and diffusely distributed glands, suggesting morphological alterations induced by PST (Figure 2).

**Figure 3:**
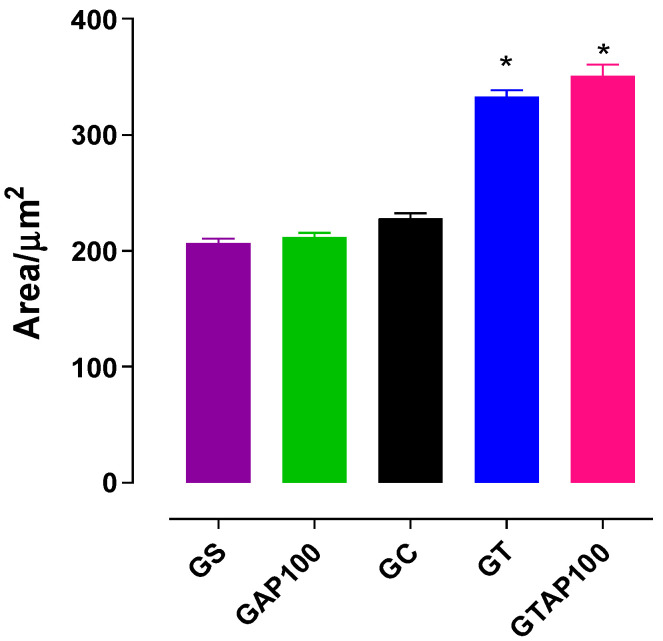
Effects of supplementation with *Arthrospira platensis* in the GS, GAP100 groups, and progressive strength training and supplementation with *A. platensis* in the GC, GT, and GTAP100 groups on the muscular area in the rat uterus. Symbols and vertical bars represent the mean and s.e.m, respectively (*n* = 5). * *p* < 0.05 (GT and GTAP100 vs. GC). GC = control group; GS = saline group; GT = trained group; GAP100 = group supplemented with *A. platensis* at a dose of 100 mg/kg; GTAP100 = group trained and supplemented with *A. platensis* at a dose of 100 mg/kg.

Morphometric analysis of the myometrial layer showed no significant difference in muscle thickness between the GS (206.7 ± 3.8 µm^2^) and GAP100 (211.7 ± 3.6 µm^2^) groups. However, GT rats demonstrated a significant increase in myometrial thickness (333.0 ± 5.5 µm^2^) compared to GC (227.8 ± 4.6 µm^2^), indicating PST-induced hypertrophy. Supplementation with *A. platensis* did not reverse this increase in GTAP100 rats (351.2 ± 9.6 µm^2^) (Figure 3). 

### 2.2. Effect of PST and A. platensis on KCl-Induced Contractility in the Presence of L-NAME

**Figure 4:**
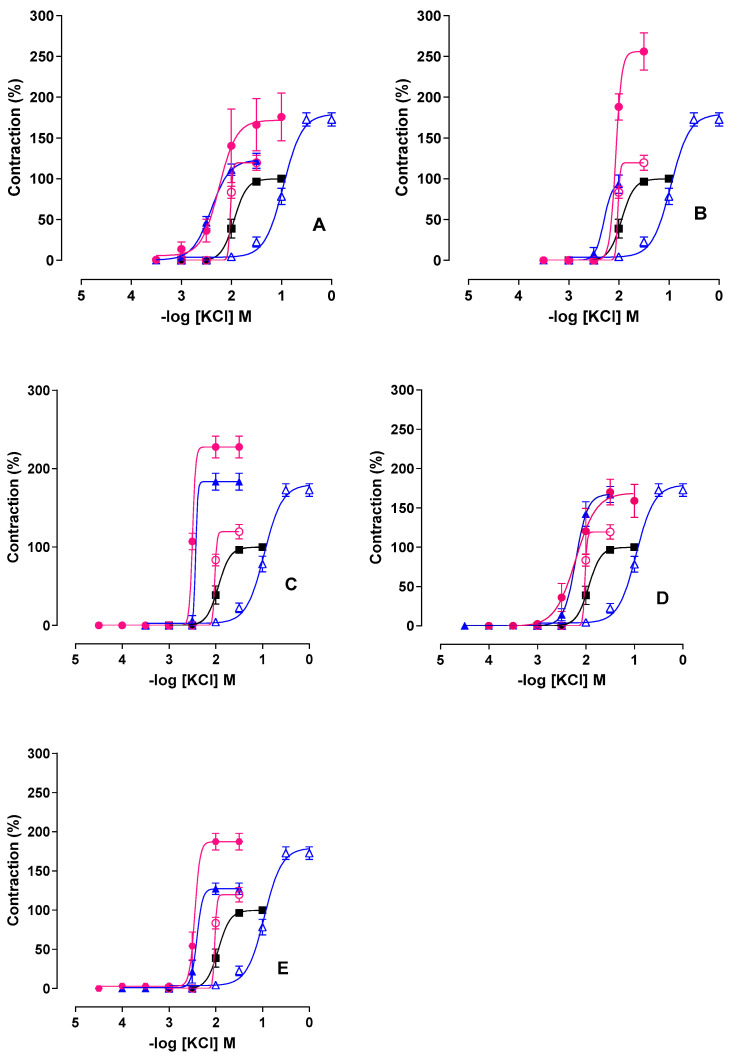
Effect of progressive strength training and/or supplementation with *A. platensis*, in the presence and/or absence of inhibitors, on the cumulative KCl dose–response curves in isolated rat uterus. (**A**) L-NAME; (**B**) indomethacin; (**C**) L-NAME + indomethacin; (**D**) apocynin; (**E**) tempol. Symbols and vertical bars represent mean ± s.e.m. (*n* = 5). (**A**) GC (■), GT (Δ), GTAP100 (◯), GT + L-NAME (▲), GTAP100 + L-NAME (⬤); (**B**) GC (■), GT (Δ), GTAP100 (◯), GT+ indomethacin (▲), GTAP100 + indomethacin (⬤); (**C**) GC (■), GT (Δ), GTAP100 (◯), GT+ L-NAME + indomethacin (▲), GTAP100 + L-NAME + indomethacin (⬤); (**D**) GC (■), GT (Δ), GTAP100 (◯), GT + apocynin (▲), GTAP100+ apocynin (⬤); (**E**) GC (■), GT (Δ), GTAP100 (◯), GT + tempol (▲), GTAP100 + tempol (⬤). The x- and y-axis scales were kept identical across all graphs to allow direct comparison of responses between experimental groups. GC = control group; GT = trained group; GT + inhibitor = trained group in the presence of inhibitor; GTAP100 = trained and supplemented group (100 mg/kg *A. platensis*); GTAP100 + inhibitor = trained and supplemented group (100 mg/kg *A. platensis*) in the presence of inhibitor.

In GT animals, the presence of L-NAME (NOS inhibitor) shifted the KCl concentration–response curve to the left and decreased efficacy by approximately 50% (Emax = 122.1 ± 9.1%; pCE_50_ = 2.4 ± 0.05) compared to the absence of inhibitor (Emax = 172.7 ± 8.1%; pCE_50_ = 1.0 ± 0.03) (Figure 4, Table 1). In GTAP100 rats, L-NAME abolished the protective effect of AP, resulting in a marked increase in contractile efficacy (Emax = 203.0 ± 27.8%) without significant change in potency (pCE_50_ = 2.1 ± 0.2).

### 2.3. Effect of PST and A. platensis in the Presence of Indomethacin

The COX inhibitor indomethacin significantly reduced KCl efficacy in GT rats (Emax = 93.4 ± 11.3%; pCE_50_ = 2.2 ± 0.06), compared to the untreated condition (Emax = 172.7 ± 8.1%; pCE_50_ = 1.0 ± 0.03) (Figure 4, Table 1). In GTAP100 rats, indomethacin markedly increased contractile efficacy (Emax = 256.0 ± 22.9%), suggesting involvement of prostanoid pathways in AP-mediated protection (Figure 4 and Table 1, *n* = 5).

### 2.4. Combined Effect of L-NAME and Indomethacin

Simultaneous administration of L-NAME and indomethacin in GT rats maintained high contractile efficacy (Emax = 183.5 ± 10.85%) and significantly increased KCl potency (pCE_50_ = 2.3 ± 0.05) compared to GT alone (Figure 4, Table 1). In the GTAP100 group, co-incubation led to even higher efficacy (Emax = 227.8 ± 14.0%; pCE_50_ = 2.5 ± 0.005), indicating synergistic modulation of NO and COX pathways by AP.

### 2.5. Effect of NADPH Oxidase Inhibition with Apocynin

In GT rats, apocynin shifted the KCl curve to the left (pCE_50_ = 2.2 ± 0.06) but had minimal effect on efficacy (Emax = 167.5 ± 10.3%), indicating ROS involvement (Figure 4, Table 1). In the GTAP100 group, apocynin increased KCl efficacy to 170.0 ± 16.3% without changing potency, suggesting AP competes with NADPH oxidase-derived ROS signaling.

### 2.6. Effect of Tempol (SOD Mimetic) on Contractility

Tempol significantly reduced KCl efficacy in GT rats (Emax = 127.2 ± 7.3%) and increased potency (pCE_50_ = 2.3 ± 0.06), compared to untreated GT (Figure 4, Table 1). In GTAP100 rats, tempol further enhanced both efficacy (Emax = 187.3 ± 10.6%) and potency (pCE_50_ = 2.4 ± 0.06), suggesting an additive antioxidant effect with AP supplementation.

These results confirm that PST enhances uterine contractility through oxidative and inflammatory mechanisms, and that *Arthrospira platensis* supplementation modulates these effects via nitric oxide, prostanoid, and ROS pathways.

## 3. Discussion

This study demonstrated that progressive strength training (PST) induces not only uterine hypertrophy but also histomorphological alterations in female rats. The increase in uterine contractility observed likely involves nitric oxide (NO) production and its interaction with reactive oxygen species (ROS), resulting in peroxynitrite formation. This process appears to activate the PLA_2_ pathway and stimulate the synthesis of contractile prostanoids, in addition to an inhibitory crosstalk between the COX and NO pathways, increased superoxide anion production via NADPH oxidase, and possibly decreased superoxide dismutase (SOD) activity.

Supplementation with *Arthrospira platensis* did not inhibit PST-induced myometrial hypertrophy. This finding suggests that myometrial thickening represents a compensatory structural adaptation to repeated contractile and hemodynamic stress during training. Such hypertrophy should not necessarily be interpreted as a pathological dysfunction but rather as part of the uterine remodeling process induced by exercise. In contrast, the protective role of *Arthrospira platensis* was manifested by preventing oxidative stress, preserving endometrial architecture, and normalizing contractile hyperreactivity. Thus, the alga did not directly block hypertrophic remodeling but mitigated the functional and molecular dysfunctions associated with PST. The underlying electromechanical mechanism seems to involve positive modulation of the NO pathway, release of relaxant prostanoids, and activation of crosstalk between these pathways. The antioxidant properties of AP may act by neutralizing ROS with contractile effects on uterine smooth muscle, as previously demonstrated in other smooth muscle tissues [32,33,34,35,36].

Physical training is known to induce morphological, metabolic, and functional adaptations in various biological systems, thereby enhancing performance and overall health. However, when improperly prescribed or combined with caloric restriction, it may cause muscle damage and elevate ROS levels, which can lead to hypothalamic dysfunction, altered gonadotropin release, menstrual disorders, infertility, and even osteoporosis [10,37,38,39,40,41,42]. Despite the importance of these consequences for female reproductive health, few studies have assessed the impact of physical training on uterine contractility [31].

Animal models are essential for understanding these mechanisms. Prior studies from our group have demonstrated that AP supplementation in rats undergoing PST improves physical performance and reduces muscle damage, oxidative stress, and inflammation [33,40]. Specifically, in the uterus, Ferreira et al. [31] showed that AP at doses of 50 and 100 mg/kg did not alter contractile potency in sedentary rats, but significantly reduced KCl-induced contractile efficacy. Moreover, when combined with PST, AP supplementation prevented the increase in contractility and decrease in potency induced by training, with 100 mg/kg proving more effective.

The uterus, due to its specific contractile patterns and sensitivity to oxidative stress, is particularly affected by PST. In our study, rats in the trained group (GT) presented with individualized and diffusely distributed endometrial glands, in contrast to the circular and preserved morphology observed in other groups. This suggests that PST, even at early stages (within 2 weeks), can induce structural alterations in the uterus that may compromise reproductive function. These changes may result from reduced levels of FSH, LH, and progesterone, which have been reported in female rats following resistance training [41,42].

It is also important to emphasize the physiological relevance of the “water-jump” protocol adopted in this study. Although this model does not directly apply mechanical load to the uterus, it is widely validated to reproduce the systemic stress of resistance training in rodents, including cardiorespiratory, metabolic, and redox adaptations. Repetitive jumping in water induces marked increases in intra-abdominal pressure and sympathetic activation, which promote redistribution of blood flow toward active skeletal muscles and transiently reduce uterine perfusion. Previous studies have demonstrated that increased intraperitoneal pressure reduces cardiac output and visceral blood flow in rodents [43], while forced swimming and diving paradigms trigger reflex vasoconstriction and redistribution of circulation [44]. In pregnant models, exercise has been shown to modulate angiogenic imbalance under reduced uterine perfusion, confirming that systemic hemodynamic stress can affect uterine physiology [45,46]. Together, these findings support the mechanistic rationale that transient ischemia and hypoxia induced by water-jump training can mimic the uterine hemodynamic and redox alterations observed during high-intensity resistance exercise in women.

Oxidative stress-induced proliferation of endometrial cells can also activate the mitogen-activated protein kinase (MAPK) pathway and ERK1/2, contributing to increased uterine contractility [47]. The observed increase in myometrial thickness in GT rats may explain the enhanced KCl contractile efficacy and decreased potency previously reported by Ferreira et al. [31]. This effect may reflect an increase in smooth muscle fiber content [48], coupled with oxidative imbalance and p38-MAPK pathway activation, which promotes smooth muscle growth [49].

Exercise-induced inflammation plays a critical role in tissue remodeling, involving local and systemic release of pro-inflammatory cytokines [50,51]. Under physiological conditions, NO is continuously produced in small amounts by endothelial nitric oxide synthase (eNOS), exerting vasodilatory effects. However, during injury or inflammation, inducible nitric oxide synthase (iNOS) is upregulated and produces large quantities of NO, which can react with ROS to form peroxynitrite, which is a cytotoxic and contractile molecule [38,52].

In our study, the non-selective NOS inhibitor L-NAME abolished the PST-induced increase in contractility in the GT group, suggesting that iNOS-derived NO reacts with superoxide to form peroxynitrite, enhancing uterine contractile responses. In contrast, in AP-supplemented trained rats (GTAP100), this protective effect was lost in the presence of L-NAME, indicating that AP may enhance eNOS phosphorylation or expression via phycocyanin, which is a key antioxidant pigment in *Arthrospira* [40,53], thereby increasing NO bioavailability.

Exercise also activates PLA_2_, which in turn stimulates NADPH oxidase activity and ROS generation in muscle mitochondria and cytosol [54,55,56]. Using indomethacin, a non-selective COX inhibitor, we observed that PST-induced increases in uterine contractility were attenuated, suggesting that COX activation and synthesis of contractile prostanoids, such as PGF_2_α, are involved [57,58].

In trained rats supplemented with AP, indomethacin abolished the preventive effect of the alga, indicating that AP modulates COX activity, possibly shifting the prostanoid balance toward relaxant mediators such as PGE_2_ [59,60]. This may be attributed to *Arthrospira* content of γ-linolenic, linoleic, arachidonic, and eicosapentaenoic acids—precursors of relaxant prostanoids [61,62]—as well as to the COX-2 inhibitory properties of phycocyanin [63].

Crosstalk between the NO and prostanoid pathways is well described in the literature. Activation of cPLA_2_ increases intracellular calcium, enhancing membrane permeability, and stimulating NO production via eNOS and neuronal NOS (nNOS) [64]. Under basal conditions, NO favors the synthesis of relaxant prostanoids, whereas its inhibition favors contractile mediators [65]. S-nitrosylation and peroxynitrite-mediated COX activation may also be involved [66,67,68].

When L-NAME and indomethacin were administered simultaneously, the PST-induced loss of KCl potency was reversed, but contractile efficacy remained high. This supports the hypothesis of an inhibitory crosstalk between the NO and COX pathways. In the GTAP100 group, however, simultaneous inhibition led to a 3.5-fold increase in KCl efficacy, suggesting that AP enhances the activation of this crosstalk in favor of relaxant signaling pathways such as NO and PGE_2_ [6,65,66,67,68,69]. We further investigated the role of ROS, particularly superoxide anion and hydrogen peroxide, in modulating uterine contractility during PST. Apocynin, an NADPH oxidase inhibitor, restored KCl potency in GT rats but did not affect contractile efficacy, confirming the role of NADPH oxidase-derived ROS in PST-induced uterine hyperreactivity [38,70,71,72].

Thus, it was evaluated whether the alterations in uterine contractile reactivity of rats subjected to PFT or to its combination with algae supplementation were correlated with the modulation of the crosstalk between the NO and prostanoid pathways, through simultaneous incubation with L-NAME and indomethacin, followed by a contraction–response curve induced with KCl.

Interestingly, in AP-supplemented rats, apocynin abolished the protective effects of the alga, suggesting that AP may compete for the same NADPH oxidase inhibition sites. Alternatively, increased cGMP induced by AP might inhibit NADPH oxidase activity and restore NO availability [73].

Finally, using tempol, an SOD mimetic, we observed that it prevented the increase in KCl-induced efficacy in GT rats while enhancing potency, supporting the detrimental role of superoxide in uterine contractility [33]. In the GTAP100 group, tempol enhanced both efficacy and potency, possibly due to the antioxidant constituents of AP (e.g., phycocyanin, β-carotene), which may act downstream of superoxide formation and promote hydrogen peroxide detoxification via SOD and glutathione reductase [63,74,75]. However, high antioxidant doses may blunt exercise-induced upregulation of endogenous enzymes such as SOD and glutathione peroxidase [76,77].

Altogether, these findings suggest that AP supplementation exerts protective effects on uterine physiology during PST by mitigating oxidative damage, restoring NO signaling, promoting relaxant prostanoid synthesis, inhibiting NADPH oxidase activity, and enhancing antioxidant defenses. These results support further investigation of AP as a functional nutraceutical for female reproductive health under physical stress conditions.

A limitation of the present work is that the signaling pathways involved were investigated using a functional pharmacological approach with specific pathway inhibitors (L-NAME, indomethacin, apocynin, and tempol). Although this strategy is classical in smooth muscle pharmacology and provides robust mechanistic evidence, it does not directly demonstrate alterations in protein expression or gene transcription. We therefore acknowledge that this is a limitation of the present study. Future investigations employing molecular biology techniques such as Western blotting, qPCR, or immunohistochemistry are required to confirm changes in NO synthase isoforms, COX enzymes, and NADPH oxidase subunits in uterine tissue. Such complementary approaches will provide more precise mechanistic insight into how *Arthrospira platensis* modulates redox and signaling pathways.

## 4. Methods and Materials

### 4.1. Obtaining and Preparing Arthrospira Platensis

Lyophilized *Arthrospira platensis* powder (Lot No. 405894) was obtained from Infinity Pharma (Hong Kong, China) and certified and fractionated by Roval Manipulação (João Pessoa, Brazil). The powder was freshly dissolved in 0.9% of NaCl saline solution before administration at a dose of 100 mg/kg. Supplementation was administered via oral gavage once daily, 30 min prior to each training session, for a total duration of 8 weeks [37].

### 4.2. Animals and Experimental Groups

Virgin female Wistar rats (*Rattus norvegicus*), aged 8 weeks and weighing between 150 and 250 g, were obtained from the Animal Production Unit (UPA) at the Institute of Drug and Medicine Research (IPeFarM), Federal University of Paraíba (UFPB), Brazil. Prior to the experiments, animals were housed under controlled conditions (21 ± 1 °C, 12 h light/dark cycle, lights on from 6:00 a.m. to 6:00 p.m.) with unrestricted access to a standard pellet diet (Nuvilab^®^, Quimtia S.A., São Paulo, Brazil) and water. To minimize hormonal variability, only virgin females of the same age range were included, and housing conditions were maintained to reduce natural estrous oscillations. Additionally, all animals were hormonally synchronized by administration of diethylstilbestrol (1 mg/kg, s.c.) 24 h before euthanasia, ensuring that uterine tissues used for functional assays were collected under the same estrous stage.

All procedures complied with institutional ethical standards and were approved by the UFPB Animal Ethics Committee (Protocol No. 5191200320, ID 000946).

Animals were randomly allocated into five groups:

1. Sedentary control (GS): Did not undergo either adaptation or training and remained completely sedentary, receiving only saline by gavage.
2. Sedentary supplemented with *A. platensis* (GAP100, 100 mg/kg): Did not undergo exercise intervention or an adaptation period and received *A. platensis* at 100 mg/kg by gavage.
3. Adapted control (GC): Underwent the one-week adaptation protocol only, which consisted of light water-jumping sessions with reduced overload (50% of body weight) and limited repetitions. This adaptation was designed to familiarize animals with the aquatic environment and apparatus, minimize stress, and prevent confounding acute effects of exercise initiation. Importantly, GC animals did not undergo subsequent progressive strength training, allowing the differentiation between physiological effects of the adaptation process itself and those induced by the full training program.
4. Trained (GT): Underwent the adaptation week followed by 8 weeks of progressive resistance training and received saline by gavage.
5. Trained supplemented with *A. platensis* (GTAP100, 100 mg/kg): Underwent the adaptation week followed by 8 weeks of progressive resistance training and received *A. platensis* at 100 mg/kg by gavage.

### 4.3. Progressive Strength Training Program (PST)

The animals in the progressive strength training group underwent a specific jumping program in a polyvinyl chloride (PVC) cylinder, which was 30 cm in diameter and 70 cm long, containing water. The depth of the water contained in the tanks was 50 cm, approximately equivalent to twice the length of the rat, aiming to limit the alternative of climbing until grabbing the edge of the cylinder. Before starting the exercise, the water was heated to a temperature of around 32 °C, as it is considered neutral in relation to the rat’s body temperature [37].

Strength training was based on a protocol of jumping in water, consisting of 4 sets of 10 to 12 repetitions, with a 30-second interval between sets, with progressive overload adjusted according to the animal’s weight. The overload was applied to the animals’ thorax through a special vest that allowed them to perform the jumps without the load disconnecting from the body or impeding their movements. An overload corresponding to the weight of the vest when wet (25 g) was considered and deducted from the specific load corresponding to the animal’s body mass for better training precision. Exercise sessions were always carried out from 12 pm to 2 pm. During each exercise series, the time spent by the animal to perform the exercise was analyzed with the aim of evaluating the effectiveness of the exercise on muscle performance.

Strength training was developed as follows: (a) adaptation week—the first week of training was intended for the adaptation of the animals; for this, the animals performed three alternating exercise sessions, with an overload equivalent to 50% of the animal’s body mass, with the number of sets and repetitions adjusted daily and a 30-second rest interval between sets. 1st day: 2 sets × 5 jumps; 2nd day: 4 sets × 5 jumps; 3rd day: 4 sets × 9 jumps. After the adaptation week, training was performed 3 days a week (alternate days) as follows: (a) 1st and 2nd weeks—the animals performed 4 sets of 10 jumps, with 30 s of rest between sets and an overload equivalent to 50% of the animal’s body weight; (b) 3rd and 4th weeks—4 sets of 10 jumps were performed, with 30 s of rest between sets and an overload of 60% of the body weight; (c) 5th and 6th weeks—the 4 sets of 10 jumps remained, with 30 s of rest between sets, but with an overload of 80% of the animal’s body weight; (d) 7th and 8th weeks—the 4 sets, 30 s of rest between sets, and an overload of 80% of the animal’s body weight remained, but with 12 jumps performed (Table 2).

### 4.4. Reagents and Solutions

All chemicals were of analytical grade. Calcium chloride dihydrate, sodium bicarbonate, potassium chloride, magnesium chloride dihydrate, glucose, sodium chloride, hydrochloric acid, and sodium hydroxide were purchased from Êxodo Científica (João Pessoa, Brazil). Diethylstilbestrol was obtained from Cayman Chemical (Ann Arbor, MI, USA). Tempol, apocynin, L-NAME (Nω-Nitro-L-arginine methyl ester hydrochloride), and indomethacin were purchased from Sigma-Aldrich (São Paulo, Brazil). Ketamine and xylazine were obtained from commercial veterinary suppliers (ketamine hydrochloride and xylazine hydrochloride).

### 4.5. Nutrient Solutions

The nutrient solution used was Locke–Ringer [78], adjusted to pH 7.4 with 1 N HCl or NaOH, continuously bubbled with carbogen (95% O_2_ and 5% CO_2_), and maintained at 32 °C. Locke–Ringer’s physiological solution was freshly prepared on the day of the experiments and had the following composition (mM): NaCl (154.0), KCl (5.6), MgCl_2_ (2.1), CaCl_2_ (2.2), glucose (5.6), and NaHCO_3_ (6.0). Solutions and incubation stocks were stored at −20 °C when appropriate.

### 4.6. Histological Analysis

Uterine and ovarian samples were fixed in 10% of neutral buffered formalin (10% NBF, with approximately 4% of *w*/*v* formaldehyde in phosphate buffer, pH 7.0–7.4) for 24–48 h at room temperature. After fixation, samples were dehydrated through an ascending ethanol series, cleared in xylene and embedded in paraffin. Dehydration and clearing steps were as follows: 70% EtOH, 5 min; 80% EtOH, 5 min; 95% EtOH, 2 × 5 min; absolute ethanol, 2 × 5 min; xylene, 2 × 10 min. Paraffin-embedding was performed using histological paraffin (melting point 56–58 °C). Embedded samples were sectioned at 4 µm thickness on a rotary microtome and mounted on glass slides.

For staining, sections were deparaffinized in xylene (2 × 10 min), rehydrated through a descending ethanol series (absolute EtOH 2 × 5 min, 95% 5 min, 80% 5 min, 70% 5 min), rinsed in distilled water, and stained with Harris hematoxylin for 1 min. Slides were then washed under running water for 5 min and stained with eosin Y (0.5% *w*/*v* in 95% ethanol) for 3 min. After staining, slides were dehydrated through the ascending ethanol series, cleared in xylene (2 × 5–10 min), and mounted with the Entellan^®^ mounting medium (Merck; ready-to-use synthetic resin mounting medium—used as supplied). The Harris hematoxylin used was a commercial Harris hematoxylin solution (11;39) [79].

### 4.7. Investigation of the Preventive Mechanism of A. platensis Supplementation on Contractile Changes Induced by Strength Training

#### 4.7.1. Preparation of Isolated Rat Uterus

The rats were treated 24 hours beforehand with diethylstilbestrol (1 mg/kg) subcutaneously for hormonal synchronization of estrus. After this time, the rats were euthanized by anesthesia with ketamine 100 mg/kg (i.p.) and xylazine 10 mg/kg (i.p.), followed by a complementary method of decapitation with the aid of a guillotine. The abdominal cavity was opened with a longitudinal cut, and the uterus was removed and cleaned of all connective and adipose tissue. Then, the two uterine horns were separated by means of an incision, opened longitudinally, and suspended vertically in bath tanks for isolated organs, containing the Locke–Ringer nutrient solution, which was heated to 32 °C and gassed with carbogen.

These tissues were kept under tension of 1 g and remained at rest for 40 min, which was the time required for stabilization of the preparation. During this period, the nutrient solution was changed every 10 min (adapted from [78]). After the stabilization period, a contraction was induced with 60 mM of KCl to verify the functionality of the organ. After 15 min, a contraction was induced with cumulative concentrations of KCl (10^−4^–10^−1^ M). The results were evaluated by comparing the amplitude of the contractile response of the uterus of the rats in the groups that received supplementation with *A. platensis* and/or were subjected to progressive strength training, with that obtained by the average of the maximum amplitudes of the control curves.

#### 4.7.2. Investigation of the Participation of the Nitric Oxide Pathway and Cyclooxygenases

##### Obtaining Cumulative Concentration–Response Curves to KCl, in the Absence and Presence of L-NAME or Indomethacin

The uterus was assembled as described in item 2.3.1 After the stabilization period, L NAME (10^−4^ M), a non-selective NOS inhibitor [80], or indomethacin (10^−5^ M), a non-selective COX inhibitor [81], was incubated for 20 min in different preparations, and then the cumulative concentration–response curves to KCl (10^−4^–10^−1^ M) were induced in the absence and presence of the inhibitors. In addition, cumulative curves to KCl were performed in other preparations in the simultaneous presence of both inhibitors.

#### 4.7.3. Investigation of the Participation of the Enzymes Nicotinamide Adenine Dinucleotide Phosphate Oxidase and Superoxide Dismutase

##### Obtaining Cumulative Concentration–Response Curves to KCl, in the Absence and Presence of Apocynin and Tempol

The uterus was assembled as described in item 2,3.1 After the stabilization period, it was incubated for 20 min with apocynin (10^−4^ M), an NADPH oxidase inhibitor [82], or tempol (10^−3^ M), an SOD mimetic [83], in different preparations and then the cumulative concentration–response curves were induced to KCl (10^−4^–10^−1^ M) in the absence and presence of the inhibitors.

### 4.8. Statistical Analysis

Data are expressed as mean ± standard error of the mean (SEM). The number of animals per experimental group is indicated in each figure legend (*n* = 5). Data were tested for normality (Shapiro–Wilk test) and homogeneity of variances. Statistical analyses were conducted using unpaired Student’s *t*-test or one-way ANOVA followed by Tukey’s post hoc test. Differences were considered significant at *p* < 0.05. Negative logarithm of the concentration of agonist that produces 50% of the maximal effect (pCE_50_) values were calculated using nonlinear regression. Emax values were defined as the maximum contractile response. Analyses were performed using GraphPad Prism^®^ 7.01 (GraphPad Software, San Diego, CA, USA).

## 5. Conclusions

The electromechanical mechanism underlying progressive strength training involves myometrial hypertrophy and is likely mediated by increased production of contractile prostanoids, NO, and superoxide anion, along with inhibitory crosstalk between the NO and COX pathways and reduced SOD activity. In female rats supplemented with *Arthrospira platensis*, PST did not prevent compensatory myometrial hypertrophy but preserved uterine function by reducing oxidative stress, preserving endometrial morphology, enhancing NO signaling, stimulating relaxant prostanoid synthesis, and inhibiting NADPH oxidase-derived ROS production. These protective effects indicate that *Arthrospira platensis* primarily modulates redox and signaling pathways rather than interfering with structural hypertrophic remodeling, thereby preventing the development of functional uterine dysfunction during resistance training. These findings suggest that *Arthrospira platensis* supplementation may serve as a promising dietary strategy to prevent oxidative damage and contractile dysregulation in the uterus induced by high-intensity strength training. If confirmed in human studies, *Arthrospira platensis* could represent a safe and effective functional supplement to support female reproductive health during resistance exercise regimens.

## Figures and Tables

**Table 1:** Emax and pCE_50_ values of KCl in isolated rat uterus, in GC, GT, GT + L-NAME, GTAP100, and GTAP100 + L-NAME.

Inhibitor	GC	GT	GT + Inhibitor	GTAP100	GTAP100 + Inhibitor
Emax (%) L-NAME			122.1 ± 9.2 ^#^		203.0 ± 27.8 *^$^
Indomethacin L-NAME + Indomethacin	100	172.7 ± 8.1 *	93.4 ± 11.3 ^#^ 183.5 ± 10.85 *	119.7 ± 9.1	256.0 ± 22.9 *^$^ 227.8 ± 14.0 *^$^
Apocynin Tempol			167.0 ± 10.3 * 127.2 ± 7.3 ^#^		170.2 ± 16.3 *^$^ 187.3 ± 10.6 *^$^
pCE_50_ L-NAME			2.4 ± 0.05 *^#^		2.1 ± 0.2
Indomethacin L-NAME + Indomethacin	2.0 ± 0.07	1.0 ± 0.03 *	2.2 ± 0.06 *^#^ 2.3 ± 0.05 ^#^	2.0 ± 0.05	2.0 ± 0.03 2.5 ± 0.005 *^$^
Apocynin Tempol			2.2 ± 0.06 ^#^ 2.3 ± 0.06 *^#^		2.2 ± 0.1 2.4 ± 0.06 *^$^

One-way ANOVA followed by Tukey’s post-test, * *p* < 0.05 (GT, GT + Inhibitor, GTAP100, and GTAP100 + Inhibitor vs. GC); ^#^ *p* < 0.05 (GT vs. GT + Inhibitor); ^$^ *p* < 0.05 (GTAP100 vs. GTAP100 + Inhibitor). GC = control group; GT = trained group; GT + inhibitor = trained group in the presence of inhibitor; GTAP100 = trained and supplemented group (100 mg/kg *A. platensis*); GTAP100 + inhibitor = trained and supplemented group (100 mg/kg *A. platensis*) in the presence of inhibitor. Data are expressed as mean ± s.e.m. (*n* = 5).

**Table 2:** Strength training protocol outline.

Adaptation (Days)			Weeks			
1st	2nd	3rd	1st and 2nd	3rd and 4th	5th and 6th	7th and 8th
2 series ×	4 series ×	4 series ×	4 series ×	4 series ×	4 series ×	4 series ×
5 jumps	5 jumps	9 jumps	10 jumps	10 jumps	10 jumps	12 jumps
50%	50%	50%	50%	60%	80%	80%

## Data Availability

The data supporting the findings of this study are available from the corresponding author upon reasonable request.

## Abbreviations

ANOVA	Analysis of variance
AP	*Arthrospira platensis*
AQP	Aquaporins
ATP	Adenosine triphosphate
CAPES	Coordination for the Improvement of Higher Education Personnel (Brazil)
CAT	Catalase
CLC3	Chloride channel 3
CNPq	National Council for Scientific and Technological Development (Brazil)
CO_2_	Carbon dioxide
COX	Cyclooxygenase
cPLA_2_	Cytosolic phospholipase A_2_
CYP450	Cytochrome P450 monooxygenases
ERK	Extracellular signal-regulated kinase
EtOH	Ethanol
FSH	Follicle-stimulating hormone
GAP100	Sedentary group supplemented with *A. platensis* (100 mg/kg)
GC	Control group
GS	Saline group
GT	Trained group
GTAP100	Trained group supplemented with *A. platensis* (100 mg/kg)
GTSP100	Trained group supplemented with *Spirulina platensis* (100 mg/kg)
HCl	Hydrochloric acid
HIIT	High-intensity interval training
IL-1β	Interleukin-1 beta
iNOS	Inducible nitric oxide synthase
IP_3_R	Inositol trisphosphate receptor
LH	Luteinizing hormone
LOX	Lipoxygenase
L-NAME	Nω-Nitro-L-arginine methyl ester hydrochloride
MAPK	Mitogen-activated protein kinase
NADPH	Nicotinamide adenine dinucleotide phosphate
NBF	Neutral buffered formalin
NO	Nitric oxide
NOS	Nitric oxide synthase
O_2_•^−^	Superoxide anion
PGE_2_	Prostaglandin E_2_
PGF2α	Prostaglandin F_2_α
PLA_2_	Phospholipase A_2_
PVC	Polyvinyl chloride
PST	Progressive strength training
qPCR	Quantitative polymerase chain reaction
RNS	Reactive nitrogen species
ROS	Reactive oxygen species
SEM	Standard error of the mean
SOD	Superoxide dismutase
UFPB	Federal University of Paraíba (Brazil)
UPA	Animal Production Unit (UFPB)
XO	Xanthine oxidase
